# Arthroscopic Posterior Capsulolabral Reconstruction With Gracilis Allograft

**DOI:** 10.1016/j.eats.2022.03.010

**Published:** 2022-06-21

**Authors:** Brett Sanders, Colton Norton

**Affiliations:** a Center for Sports Medicine and Orthopedics, Chattanooga, Tennessee, U.S.A; b University of Tennessee at Chattanooga Department of Orthopedics,Chattanooga, Tennessee, U.S.A

## Abstract

Posterior shoulder instability is increasingly recognized and represents a complex continuum of pathology that can be challenging to diagnose and treat. Current surgical options involve posterior labral repair with or without capsular plication, as well as bony procedures, including glenoid bone grafting and glenoid osteotomy when indicated, often in the setting of revision. There is presently a dearth of surgical options to directly address the root cause of soft tissue failure, including a diminutive posterior labrum, chondrolabral retroversion, and thin or hyperelastic posterior capsule. This article presents a technique for arthroscopically augmenting the posterior capsulolabral complex in the setting of soft tissue insufficiency, laxity, or poor prognostic factors for failure. Secondarily, this technique provides a lower risk revision option for reconstruction in failed posterior instability without glenoid bone defect.

## Introduction

Posterior instability was initially described in 1839 by Sir Astley Cooper.^1^ The more severe cases of unidirectional posterior dislocation associated with seizure disorders, trauma, and electrocution have been known for decades, but the surgical treatment of posterior instability has lagged behind that of anterior instability, likely due to the large range of the clinical continuum of presentation and difficulty with diagnosis on physical exam and imaging modalities of more subtle presentations. Although the percentage of frank posterior dislocations are low and account for 2-6%^2^ of all shoulder dislocations, most posterior instability comprises subluxation events or simply pain, and thus are likely underdiagnosed. This distinction was emphasized by Hawkins in 1984 and termed RPS (recurrent posterior subluxation). In the 1990’s and early 2000’s, small arthroscopic series with limited follow-up were reported,^3^^,^^9^ and Bradley documented several large series of arthroscopic posterior labral repairs beginning in 2005, recognizing the occurrence of posterior subluxations in the athletic population usually caused by microtrauma.^3^, ^4^, ^5^, ^6^, ^7^, ^8^ These authors termed their procedure reconstruction, but it appears that the technique involved a primary repair of posterior stabilizing structures without augmentation. Subsequently, other clinical studies followed, showing good results of surgical management with posterior repair.^9^ More recent assessments of the epidemiology have concurred with the clinical reports that the relative percentage of posterior instability may be much higher than traditionally thought in active populations, accounting for 43% of all shoulder instability, with 24.2% isolated posterior instability and 18.6% combined.^10^ Posterior labral repair and capsulolabral plication have been described with good results in athletes with traumatic lesions and subtle labral tears (Kim lesion). In more severe cases, bone loss may contribute to failure of posterior labral repairs. Grafting techniques have been described to address bone loss if it is thought to be the root cause of failure.^11^, ^12^, ^13^

The pathophysiology of posterior instability is at present incompletely understood and may be multifactorial. The posterior labrum and capsulolabral complex are thought to be the primary stabilizers to unidirectional instability, but other factors may also be at play. Glenoid retroversion is a known factor in refractory posterior instability, but glenoid osteotomy is technically difficult, fraught with complications such as intra-articular fracture, and has mixed results in terms of outcomes.^14^ Scapular kinematics may be impaired either as a cause of posterior instability or an adaptation to it. Thin or capacious posterior capsule, glenoid hypoplasia, or chondrolabral retroversion, diminutive labrum, MDI, hyperlaxity, or unrecognized tears may all contribute. Often, once the diagnosis is made, the patient may respond initially to labral repair, but fail or recur due to associated diagnoses. Once significant bone loss is ruled out, and especially if the patient has responded clinically to posterior labral repair, capsulolabral reconstruction may be performed as a salvage for recurrent instability with less risk for complications and future arthritis than aggressive bone procedures. Karpyshyn et al. have recently described reconstruction of the posterior capsule using acellular dermal allograft in the setting of failed repair with Ehlers-Danlos Syndrome using similar reasoning.^15^ The following technique was devised as an augment and treatment of the soft tissue causes of refractory posterior instability by a three-fold mechanism: augmentation of the diminutive posterior labrum, enhancement of the strength of the thin posterior capsule and reduction of its volume, and static recentering of the humeral head and tethering to the scapula to aid in scapular dynamic rehabilitation and proprioception.

### Patient Evaluation, Imaging, and Indications

Patients typically complain of posterior pain or instability accompanied by a positive O’Briens test, Jerk or crank test, and positive posterior translation on stability exam. Many patients may have MDI or hyper laxity findings on exam such as a sulcus sign, thumb to forearm sign, and hyperextensile elbows. Scapular dyskinesia may be present.

### Imaging

Plain radiographs are usually negative. MRI with gadolineum may show a frank posterior labral tear, Kim lesion, capacious posterior capsule, or chondrolabral retroversion.

### Indications

The procedure is currently indicated for revision of previously diagnosed posterior instability with a good response to primary posterior labral repair and capsular plication, or known soft tissue deficit of the posterior stabilizing structures (thin or deficient posterior capsule/diminutive labrum). These indications could be expanded to individuals with high risk for primary failure due to hyperlaxity.

## Surgical Technique

**Fig 1:**
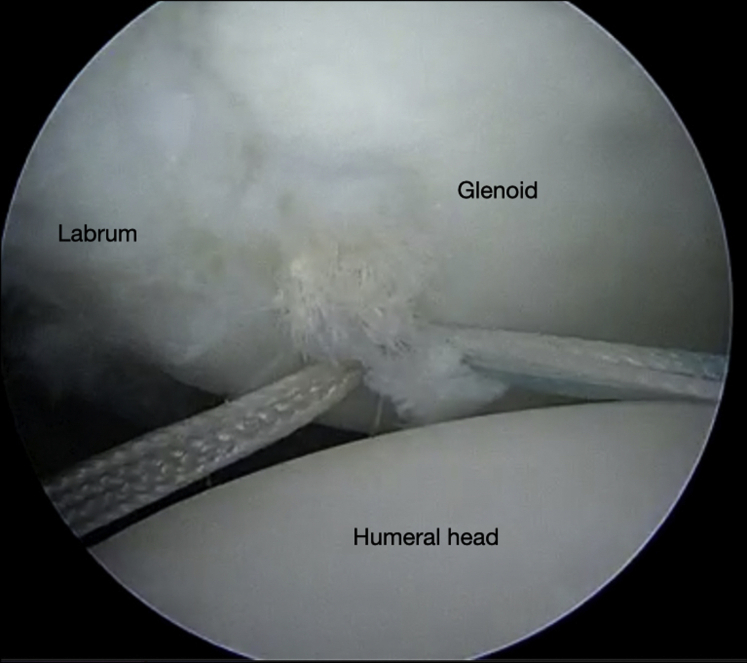
Right shoulder, beach chair position. View from superior portal showing anchor placed in 7 o’clock position.

**Fig 2:**
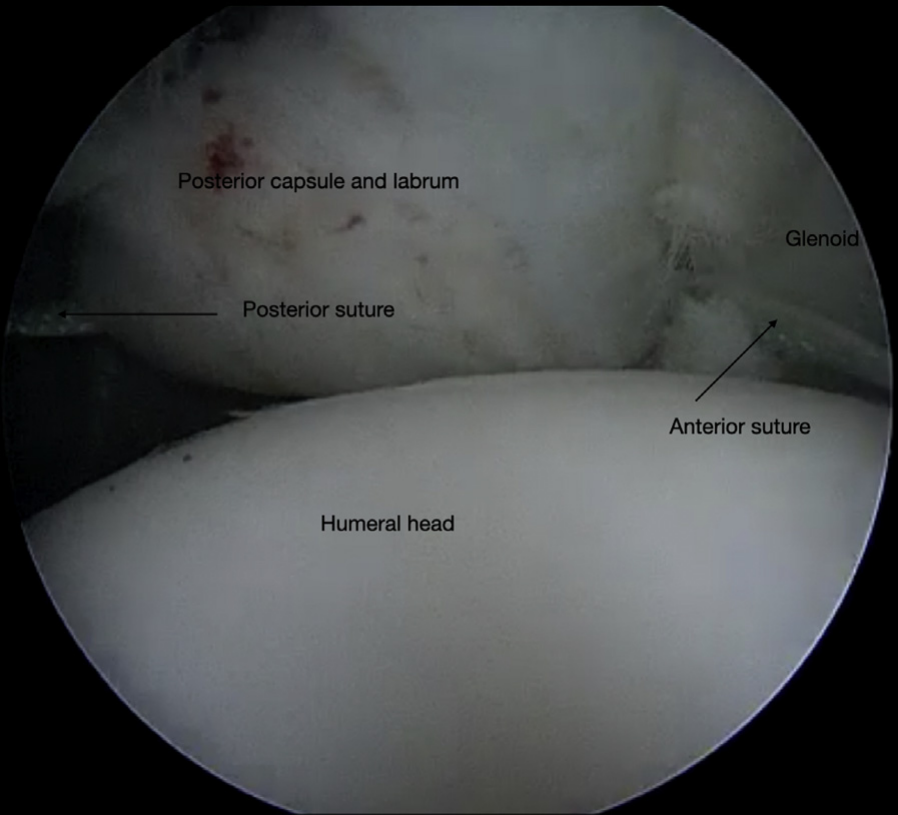
Right shoulder, beach chair position. View from superior portal showing suture passed through capsule.

**Fig 3:**
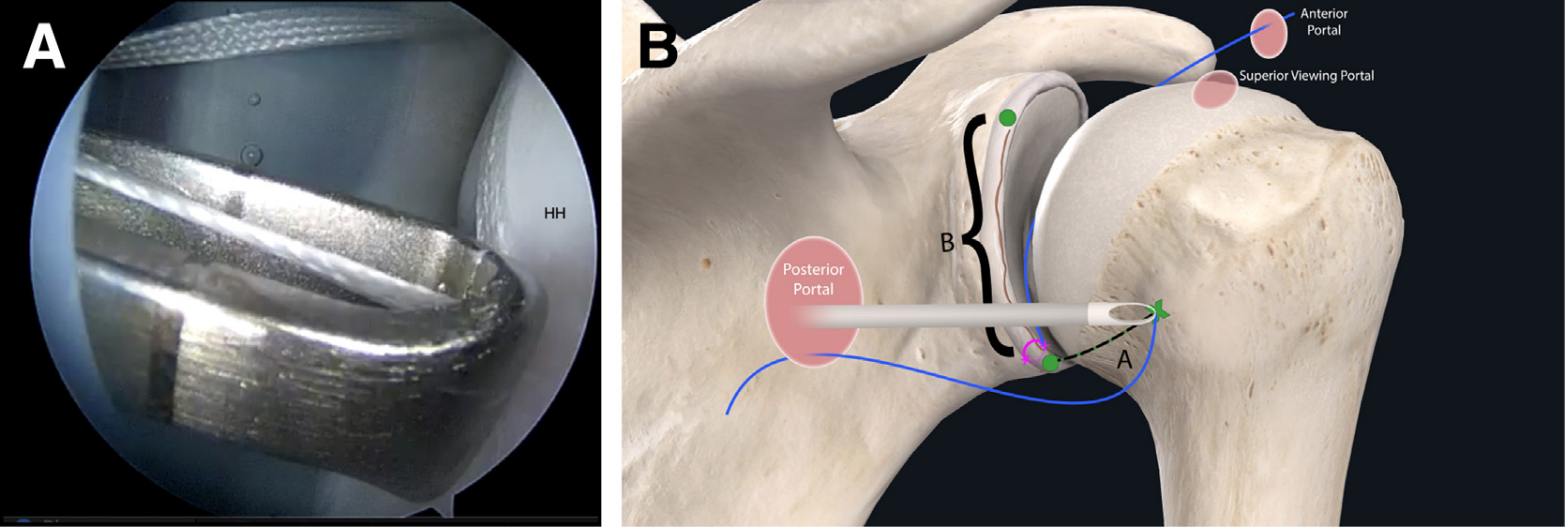
Right shoulder, beach chair position. (A) View from superior portal showing measurement, which is the curvilinear distance from the anchor to the graft insertion point on the humeral head (HH). (B) Schematic of shoulder from posterior view showing provisional labral repair and technique of using the suture to measure lengths A and B.

**Fig 4:**
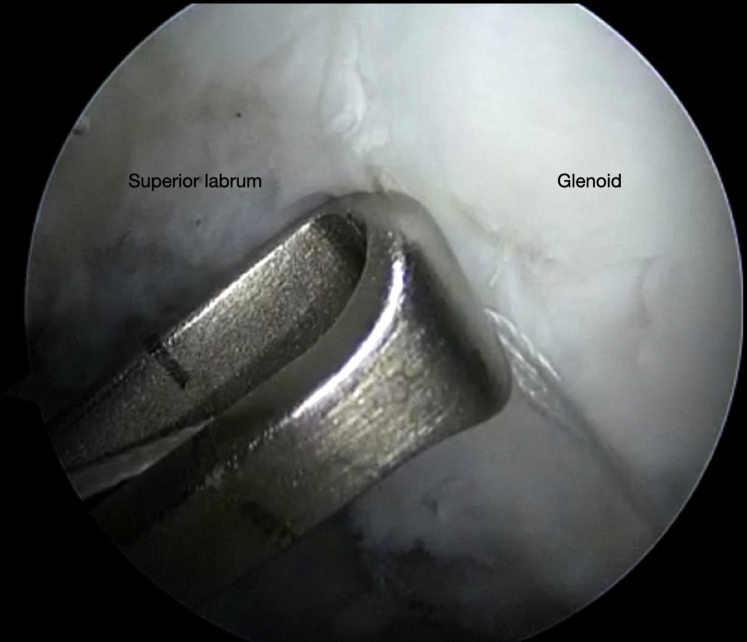
Right shoulder, beach chair position. View from superior portal showing measurement B.

**Fig 5:**
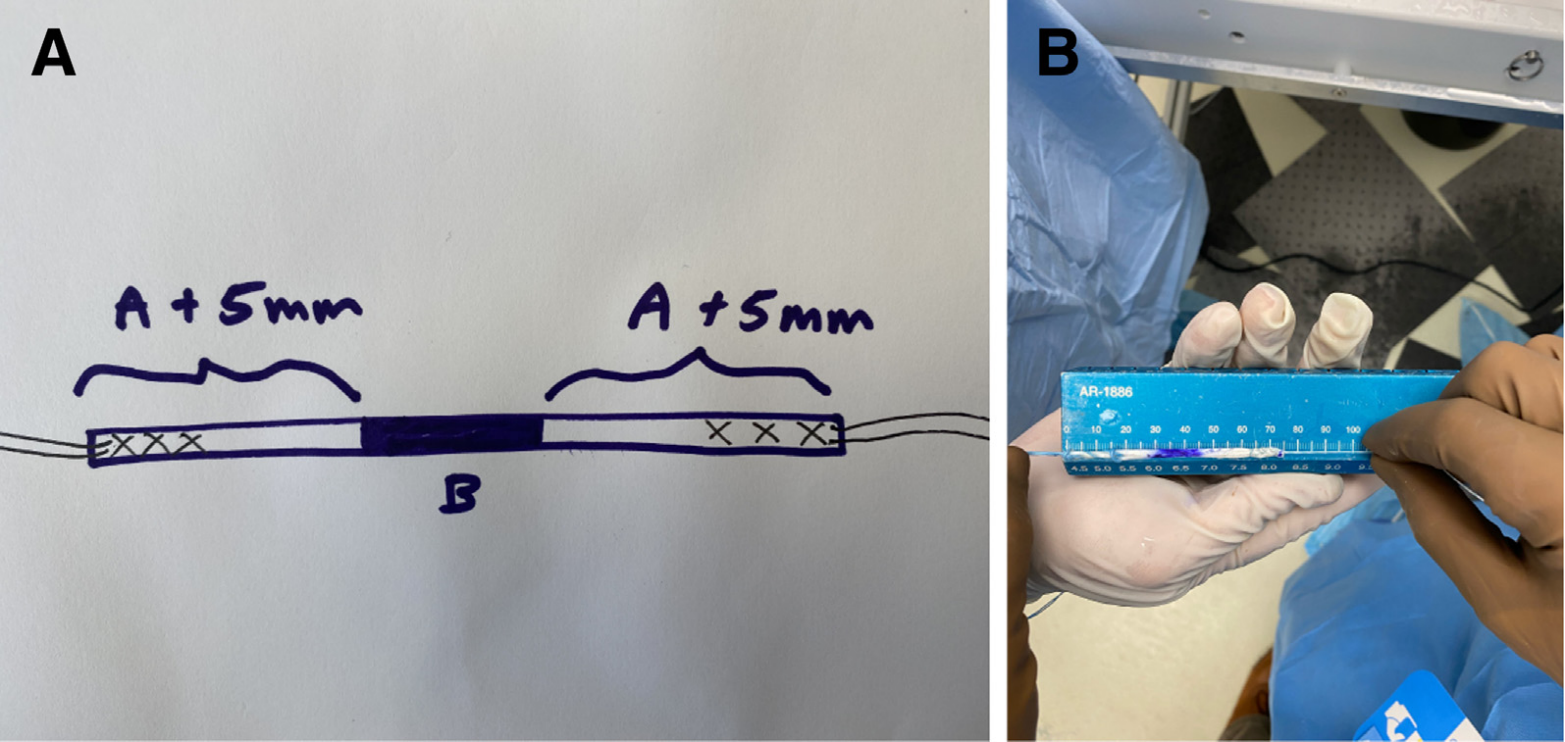
(A) Schematic of measurements applied to the graft. (B) Graft prepared with whipstitched ends on a measuring block. The middle section, measurement B, is colored with a sterile pen.

**Fig 6:**
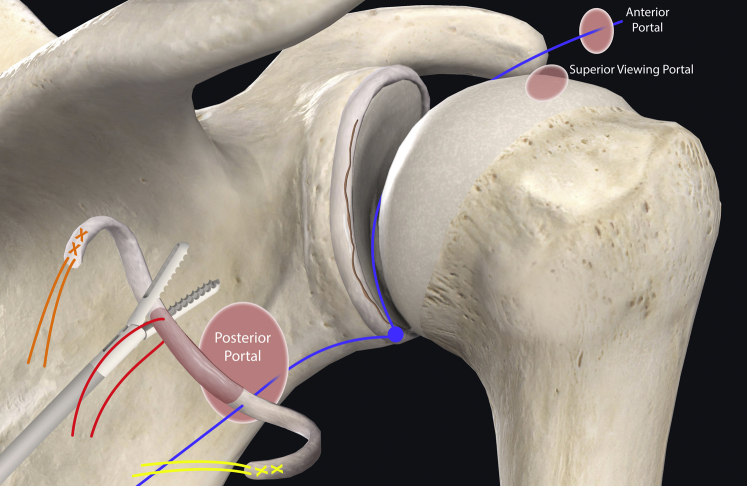
Schematic of shoulder from posterior view demonstrating the method of placing a suture tape through the inferior portion of section B, and a free suture through superior portion of section B outside the body, in preparation for graft passage.

**Fig 7:**
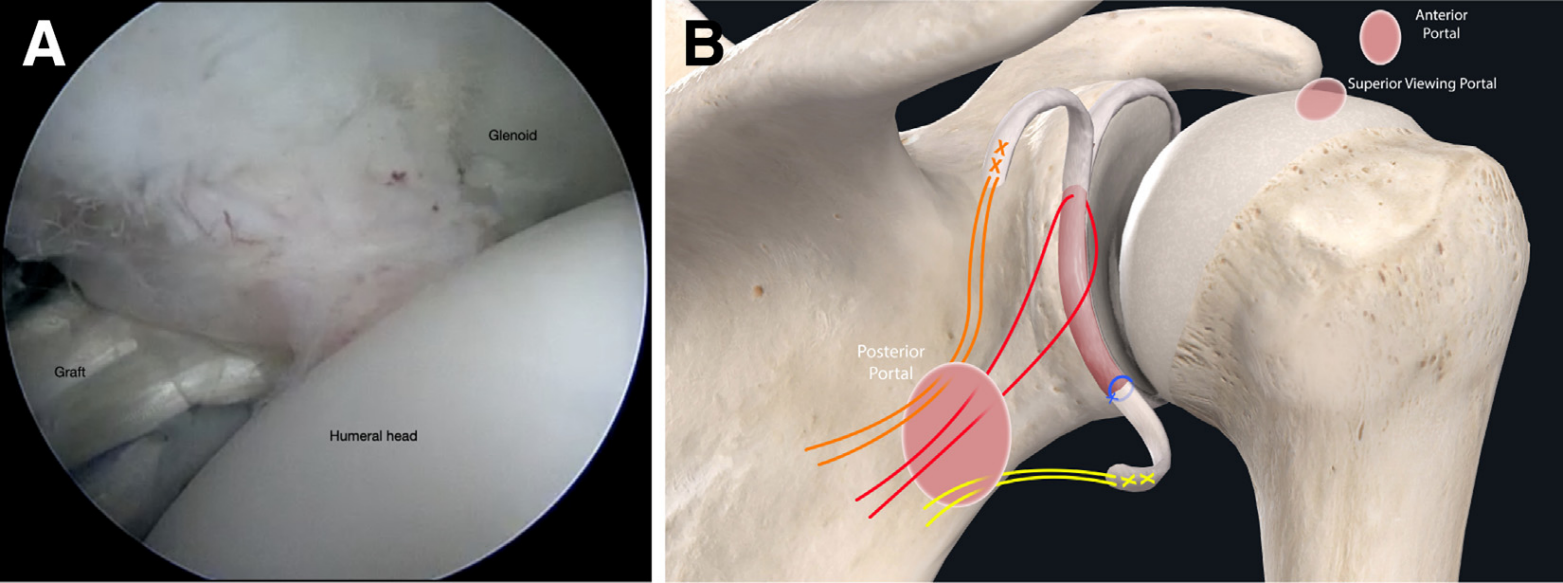
Right shoulder, beach chair position. (A) View from superior portal showing measurement inferior portion of graft tied to the suture anchor using a knot pusher. (B) Schematic of shoulder from posterior view showing inferior portion of graft secured to the glenoid. The superior free suture has not been fixed yet.

**Fig 8:**
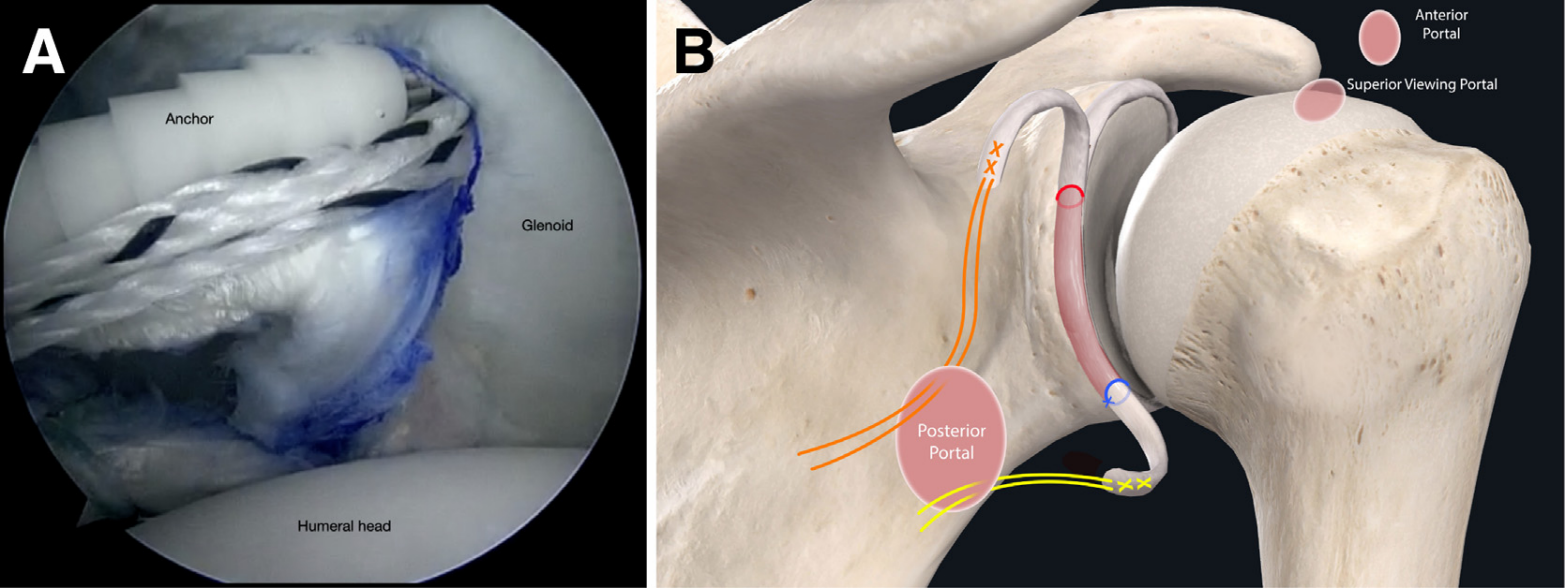
Right shoulder, beach chair position. (A) View from superior portal showing superior portion of graft being secured with a knotless suture anchor. (B) Schematic of shoulder from posterior view showing superior portion of graft fixed with a knotless anchor.

**Fig 9:**
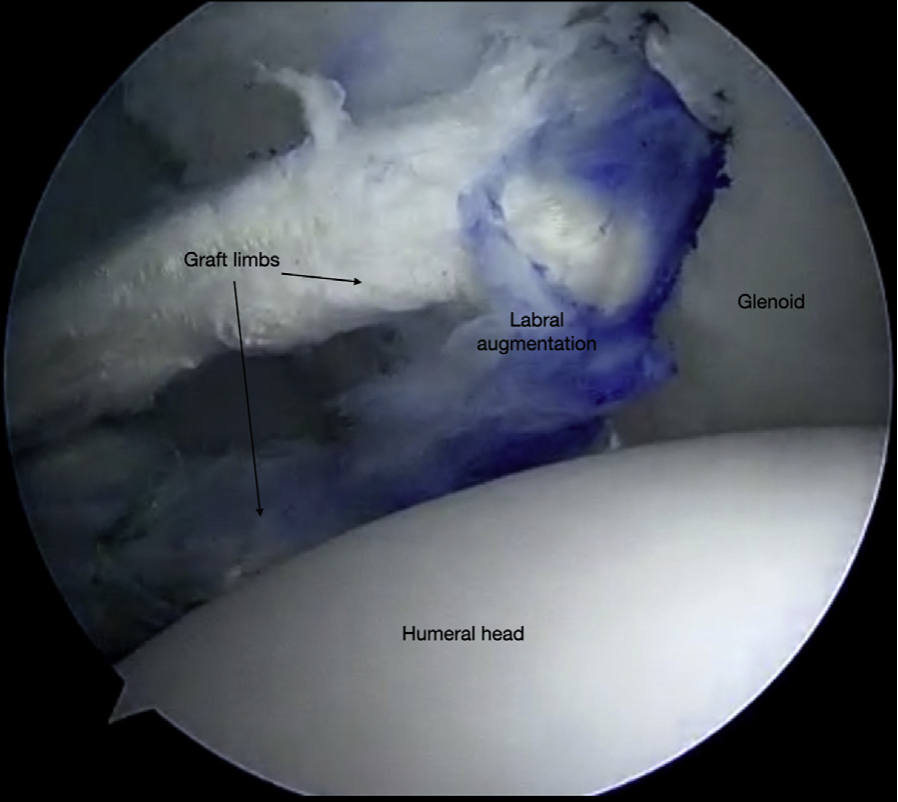
Right shoulder, beach chair position. View from superior portal showing graft secured to the glenoid with tails free and within the posterior cannula.

**Fig 10:**
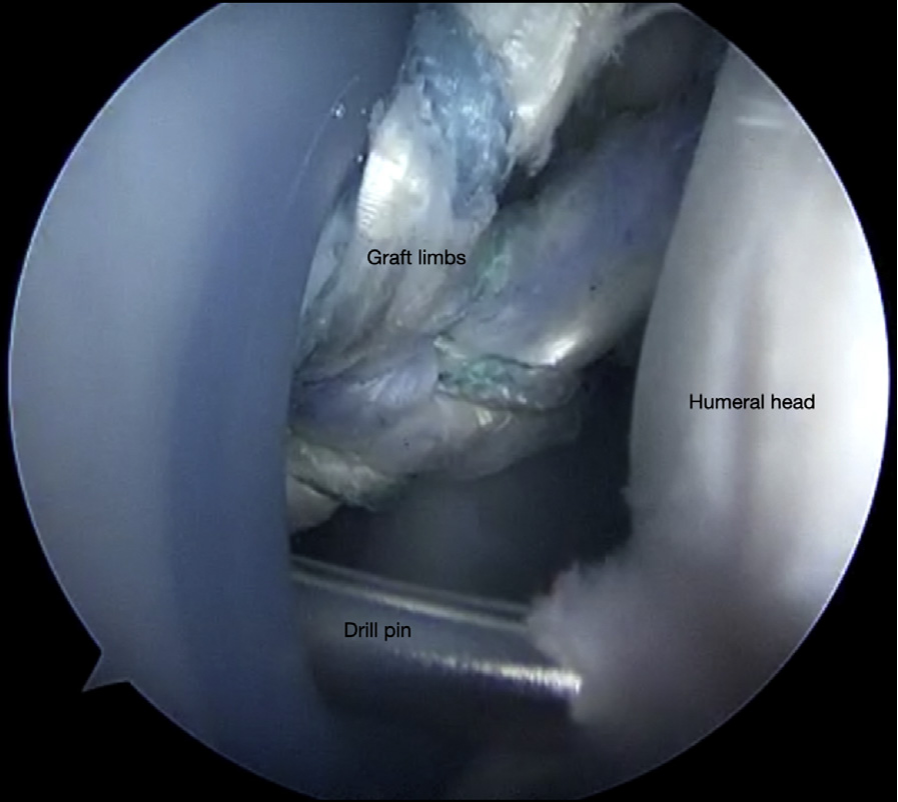
Right shoulder, beach chair position. View from superior portal showing the guide pin in place to over-ream a 25-mm socket for the graft tails.

**Fig 11:**
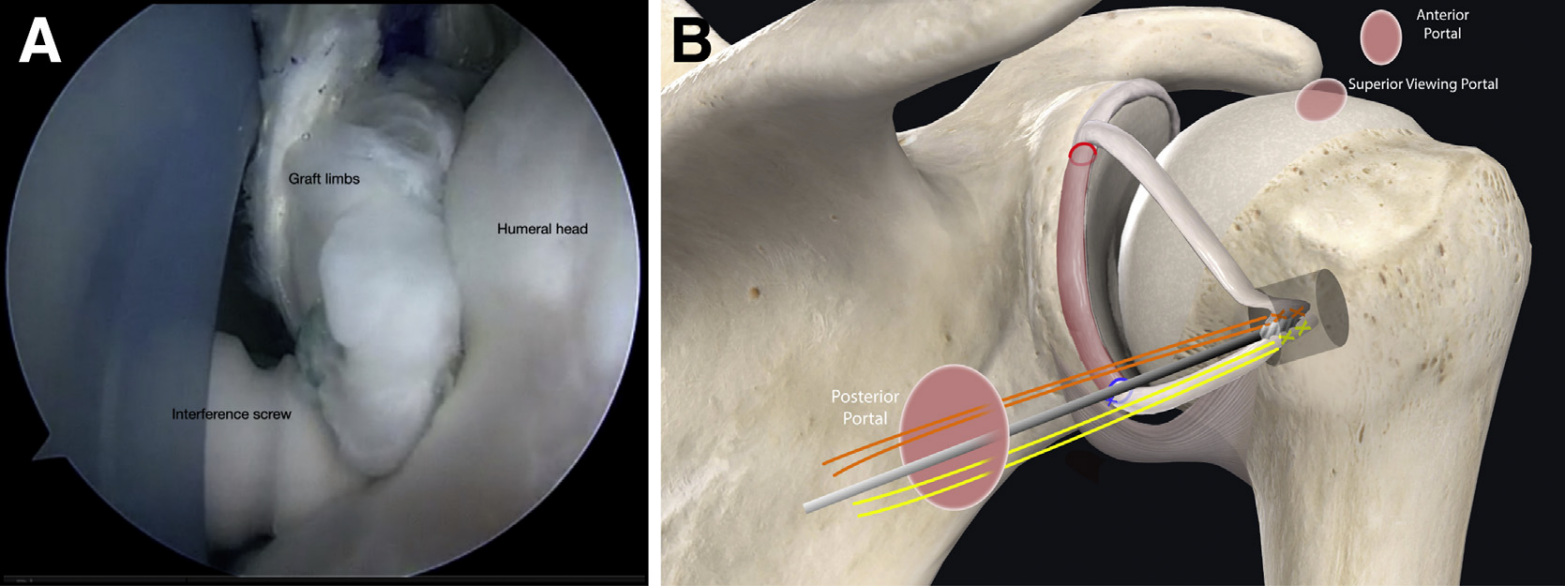
Right shoulder, beachchair position. (A) View from superior portal showing placement of biointerference screw for humeral fixation of the graft tails. (B) Schematic of shoulder from posterior view showing placement of graft tails into the socket in the humerus.

**Fig 12:**
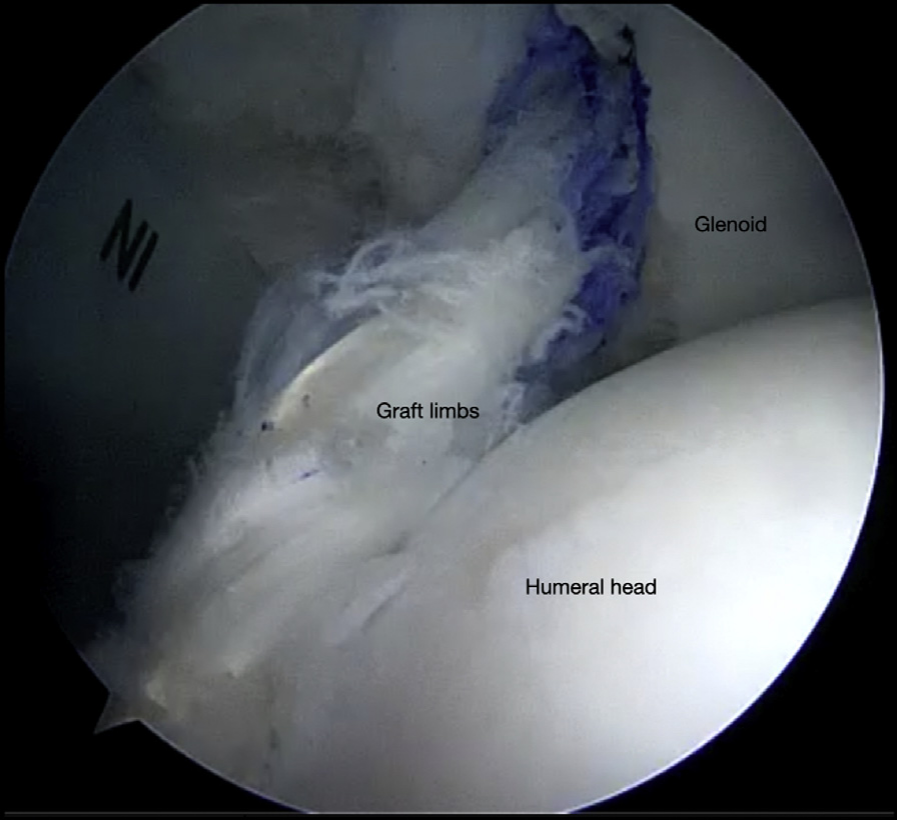
Right shoulder, beach chair position. View from superior portal showing final graft placement with secure fixation on the glenoid and humerus.

**Fig 13:**
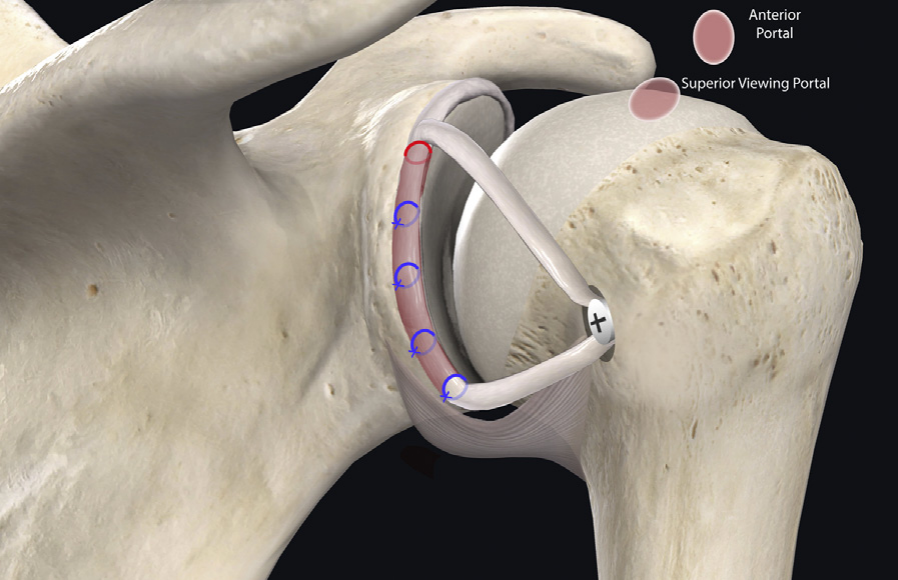
Schematic view of final graft placement in right shoulder viewing from posterior direction.

The authors prefer beach chair position for posterior instability surgery. After performing diagnostic arthroscopy, a portal of Wilmington superior viewing portal is created to allow for visualization of the posterior labrum and capsule, and the standard anterosuperior portal and posterior working portal are used (Video 1). The labrum is mobilized in the standard fashion, and a bleeding healing zone is created on the scapular neck with a motorized shaver. A double-loaded soft all-suture anchor with high-strength tapes is placed in the 7 o’clock position of the glenoid through a 10-mm passport cannula in the posterior portal, which is used for instrumentation throughout this technique (Fig 1). Standard antegrade suture-passing techniques are used to pull the first limb of suture back through the labrum and out of the posterior portal. This limb is tied to perform the preliminary anatomic labral repair. The second limb is passed through the posterior labrum and capsule (Fig 2). An arthroscopic measuring device (Arthrex, Naples, FL) is then introduced through the posterior portal and, using the second untied limb of the suture tape, a curvilinear distance is measured from the previously placed anchor in the 7 o’clock position to the bare area of the humeral head adjacent to the insertion of posterior inferior glenohumeral ligament (measurement A, Fig 3, A and B). Using the same technique, we measured the posterior glenoid anchor position distance to the intended position of the second anchor at approximately the 11 o’clock position (measurement B, Fig 4). These measurements allow for the creation of the graft. An additional 5 mm is added to the length of measurement A to allow for graft interface into a tunnel in the humeral head. Thus, the total length of the graft will be 2A +10 mm + B. The graft ends are whipstitched with fiber loop of different colors on both ends. Length B is colored with a sterile pen to allow visualization of the sections of the graft (Fig 5, A and B). A limb from the inferior anchor is brought out of the posterior cannula and placed at the inferior portion of the colored section of the graft. A free suture is placed through the superior portion of section B (Fig 6). The graft is inserted, by pushing into the joint with a knot pusher and tying a static knot at the inferior anchor (Fig 7, A and B). The second portion of the graft is pushed into the joint and fixed with a knotless anchor under appropriate tension (Fig 8, A and B). The boundaries of the labral augmentation are now achieved, with both limbs of the graft still present in the passport cannula (Fig 9). A guide pin is then placed through the passport cannula into the insertion zone of the graft on the humerus, which is just adjacent to the bare area on the posterior humerus (Fig 10). The pin is overreamed to create a 25-30-mm socket in the humerus the same diameter as the graft limbs. The previously placed suture limbs of the graft tails are then placed through the eyelet of a Swivel Lock anchor (Arthrex), and secured into the socket with an interference screw fixation with the arm held at zero degrees external rotation. (Figure 11, A and B). Further anchors can be placed into the glenoid to repair section B of the augmentation to the glenoid in the standard fashion at the discretion of the surgeon (Fig 12). The graft limbs may be incorporated into the posterior capsule to reduce volume and aid in healing. Fig 13 demonstrates a schematic of the final position of the graft from the posterior view. Table 1 lists pearls and pitfalls of the technique.

**Table 1:** Pearls and Pitfalls of Posterior Capsule Reconstruction

Pearls	Pitfalls
Accurate measurement of graft will allow less graft material to manage in the joint.	Overtightening of posterior joint during preliminary labral repair can make visualization difficult.
Use of different colored suture is recommended to avoid confusion intra-operatively.	Overconstraint is possible if graft tensioning is not performed in neutral rotation.
Gracilis is preferred over larger grafts to avoid technical difficulties placing graft.	Graft can be inappropriately positioned in joint if guiding sutures are not placed at appropriate positions as described.

### Rehabilitation

The patient is placed in an abduction sling for 6 weeks postoperatively, with rehab beginning at 4 weeks allowing passive ER and limiting IR past the hip. Active and active assist motion is permitted at 6 weeks. Strengthening begins at 3 months.

## Discussion

Posterior instability and recurrent subluxation are an increasingly recognized pathology in the shoulder with multifactorial causes. Root causes of failure are often difficult to determine, but it is likely that insufficient soft tissue elements, including labrum, capsule, and hyperlaxity are involved in a significant percentage. This technique has the advantage of providing a viable alternative to aggressive bone procedures to treat recurrent posterior instability at the capsule and labrum level with allograft augmentation, following basic principles of ligament reconstruction while leaving open all future options for more aggressive bone procedures (Table 2). In select primary case with high risk for failure, this technique adds another option to reduce failure risk.

**Table 2:** Advantages and Disadvantages of Posterior Capsule Reconstruction

Advantages	Disadvantages
Follows basic principles of soft tissue reconstruction as other techniques about the knee and shoulder, allowing graft healing in a bone socket	Technically demanding
Addresses the root cause of pathology in many cases (soft tissue insufficiency or deficit)	Incorporation rate of graft unknown
Tethers the humerus to scapula to allow stability for further rehabilitation	May not reconstruct all root causes of pathology
Augments the diminutive posterior capsule and labrum, increasing concavity-compression effect of stability	Overconstraint may be possible with loss of internal rotation
Allows a revision surgery for refractory instability that does not have complications involving the joint surface, which could result in early degenerative joint disease, such as bone procedures or osteotomies, but does not prevent further advanced reconstructive techniques	

## Supplementary Data

ICMJE author disclosure forms

Video 1 This video depicts an arthroscopic technique for reconstructing the posterior labrum and augmenting the glenohumeral posterior capsule in the setting of recurrent instability. Patient is in beach chair position and right shoulder is viewed from the superior portal.
